# Assessment of cognitive learning function in children with obstructive sleep breathing disorders

**DOI:** 10.1016/S1808-8694(15)30074-4

**Published:** 2015-10-19

**Authors:** Sandra Fumi Hamasaki Uema, Shirley Shizue Nagata Pignatari, Reginaldo Raimundo Fujita, Gustavo Antônio Moreira, Márcia Pradella-Hallinan, Luc Weckx

**Affiliations:** a Master’s degree in medical sciences - UNIFESP. Otorhinolaryngologist; b Associate professor of the Pediatric Otorhinolaryngology Discipline in the Otorhinolaryngology and Head & Neck Surgery Department - UNIFESP, Head of the Pediatric Otorhinolaryngology Discipline in the Otorhinolaryngology and Head & Neck Surgery Department - UNIFESP; c Assistant professor of the Otorhinolaryngology and Head & Neck Surgery Department - UNIFESP. Clinical unit head in the Otorhinolaryngology Discipline of the Otorhinolaryngology and Head & Neck Surgery Department - UNIFESP; d Assistant professor of the Psychobiology Department, physician of the Psychobiology Department; e Assistant professor of the Psychobiology Discipline, physician of the Psychobiology Department; f Full professor of the Otorhinolaryngology and Head & Neck Surgery Department - UNIFESP. Head of the graduation course in the Otorhinolaryngology and Head & Neck Surgery Department - UNIFESP. Sao Paulo Federal University, Pediatric Otorhinolaryngology Discipline of the Otorhinolaryngology and Head & Neck Surgery Department

**Keywords:** learning, cognition, children, obstructive sleep disorders

## Abstract

Sleep obstructive breathing disorders are frequent in children but the impact of sleep deprivation on the cognitive learning function is unclear. **Aim:** To establish whether patients with sleep obstructive breathing disorders show any functional change in learning, memory and attention. **Material and Methods:** Eighty-one children aged from 6 to 12 years were divided into 3 groups: obstructive sleep apnea syndrome (OSAS), n=24; primary snoring (PS), n=37; and control, n=20. The groups were assessed using learning (Rey) and psychological (Digit, Code, Letter Concealing, and Symbol) tests. **Results:** OSAS and PS children showed statistically significant worse performance on the variable A1 in Rey test (learning and memory) when compared with controls (p=0.011). PS children had an even worse performance on the variables A2, A4, AT and A6 compared to OSAS participants and controls (p=0.020;p=0.050;p=0.004,p= 0.05). **Conclusion:** Children with obstructive sleep breathing disorders, in particular PS, show worse Rey test scores. PS and OSAS children performed similarly in attention tests.

## INTRODUCTION

Obstructive sleep disorders (OSDs) are relatively frequent in the pediatric population, including primary snoring (PS) and the obstructive sleep apnea and hypopnea syndrome (OSAHS).^1^ The clinical picture of PS includes noisy breathing with preservation of sleep patterns, adequate alveolar ventilation and normal arterial oxygen saturation. This entity is frequent in childhood, affecting about 7 to 9% of children aged between 1 and 10 years. Some authors have estimated that the percentage of snoring in children may reach 12%, depending on age.^2^, ^3^, ^4^ There is much controversy about the definition of snoring as a benign condition as a result of studies on the sleep quality and daytime behavior of these children.5 OSAHS in childhood is characterized by snoring or noisy breathing during sleep, usually associated with hypoxemia, hypercapnia, and daytime symptoms such as oral breathing, altered behavior and excessive sleepiness. Although the incidence of this condition is not defined precisely in the literature, evidence suggests that OSAHS in childhood affects 1 to 2% of children,^6^, ^7^ with an equal gender distribution.^8^, ^9^ The medical diagnosis of OSAHS in childhood is not simple, as most children snore habitually, and the clinical history may not be sufficiently reliable to differentiate OSAHS from PS.^10^ For this reason, assessment of breathing during sleep is needed for a clear diagnosis.^11^ Since only a few children with PS develop OSAHS, and since the clinical history and physical examination usually are insufficient to establish the presence and severity of OSAHS, polysomnography becomes essential for diagnosis. When untreated, this condition may result in inadequate growth and poor neuropsychomotor development.^6^

Some studies have attempted to establish a relation between OSAHS in childhood and behavioral changes, learning difficulties, and memory and attention problems. Although excessive daytime sleepiness is an important complaint in adults with OSAHS,^12^, ^13^ few parents describe their children with OSAHS as drowsy.^14^, ^3^, ^10^ A major North-American study found OSAHS in 18% of children that had been included among the 10% worse school performer group in primary school; in these children significant school performance improvements were observed following tonsillectomy.^15^ Other studies in children with OSAHS have shown specific learning cognitive and mental processing deficits.^14^, ^16^, ^17^ Results of a major investigation conducted on 782 children have suggested that there is an association between excessive daytime sleepiness and hyperactivity, aggressive behavior, and snoring.^2^, ^18^ Still other studies have shown that psychomotor development in children is compromised if there is total sleep deprivation, but not in partial sleep deprivation.^19^

Many studies have attempted to verify the effect of sleep on memory and learning, including the effect of sleep on material that has been learnt and forgotten before and after sleep, the duration of time elapsed before the retention test and the type of material that was learnt. ^20^

Although there is a growing consensus that the prevalence of sleep deprivation in our society has increased, the impact of sleep loss on cognitive abilities has not been clearly established.^21^ Furthermore, the cognitive consequences of the interruption of sleep patterns and hypoxemia as a result of breathing disorders in children with OSAHS are still poorly defined in this group.^22^, ^23^ A study with rodents demonstrated that intermittent hypoxemia induces a substantial increase in neuron cell loss as well as adverse effects on special memory even if sleep fragmentation or deprivation is absent. ^24^

The aim of our study was to verify if children with OSDs presented altered learning, memory and attention.

## SERIES AND METHODS

This study was conducted in the Pediatric Otorhinolaryngology Discipline Outpatient Unit of the Paulista Medical School, Sao Paulo Federal University between October 2004 and September 2005. The procedures and consent terms received prior approval from the Research Ethics Committee of the Sao Paulo Federal University (protocol number 0912/04).

The sample included 81 children of both genders, mouth breathers, that fulfilled the following inclusion and exclusion criteria.

### Inclusion criteria

- children aged between 6 and 12 years with obstructive sleep disorders according to polysomnographic criteria.

### Exclusion criteria

- altered neuropsychomotor development;
- psychiatric and behavioral disorders;
- syndromes;
- altered vision;
- use of medication that acted on the central nervous system
- auditory alterations (history, otoscopy and audiometry).

The sample group was divided into 2 subgroups, as follows: patients with OSAHS and patients with PS. The control group included children of similar age in whom polysomnography did not detect OSDs.

OSAHS group: 24 patients, 10 males, 14 females.

PS group: 37 patients, 22 males, 11 females.

Control group: 20 patients, 09 males, 11 females.

Children that were included went through clinical history-taking and a general physical examination. The otorhinolaryngological exam included oroscopy, anterior rhinoscopy and otoscopy. All of the children underwent nasofibrolaryngoscopy done with a Machida (model ENT 30 P III, 3.2mm diameter) flexible fiber nasofibrolaryngoscope, a xenon light source (Styker, Othobean II), a video camera (Toshiba CCD IKM30AK) and a video monitor (Sony KV-CR). Other tests included immitance testing, and pure tone and voice audiometry (Audiometer Madsen, model Midimate 622 and earphone TDH - 39 - ANSI standard, 1969). Polysomnography was carried out to diagnose OSAHS and PS. Polysomnographies were done at the Sao Paulo Hospital Sleep Institute, at night, with the patient sleeping in a comfortable bed in a darkened and silent room. During the test, electrophysiological and cardiorespiratory parameters were recorded on a computer system (Alice 3 Healthdyne/respironics, Marrieta, GA) that included: an electroencephalogram (C3/A2, C4/A2, O1/A2 O2/A1), a submentonian and tibial electromyogram, a right and left electrooculogram, recordings of oronasal air flow, abdominal and thoracic movements, a microphone (snoring), recording of oxyhemoglobin saturation (SAO2) and the position in the bed in ambient air.

### Assessment of learning and cognitive function

I - Rey Auditory Verbal Learning Test - RAVLT), 1998 (translated by the psychologist Maria Helena da Silva Noffs).

### Variables included

- A1 (number of words recalled after a 1st reading of list A)
- A2 (number of words recalled after a 2nd reading of list A)
- A3 (number of words recalled after a 3rd reading of list A)
- A4 (number of words recalled after a 4th reading of list A)
- A5 (number of words recalled after a 5th reading of list A)
- AT (total number of words recalled: A1 to A5)
- B1 (number of words recalled after reading list B)
- A6 (number of list A words recalled spontaneously immediately after reading list B1)
- A7 (number of list A words recalled spontaneously after 20 minutes)
- R (number of correct words upon reading the recognition list)
II Coding subtest, 1991 (WISC-III - Wechsler intelligence scale for children).
* Variables included:
- total number of symbols copied correctly.
III Digit subtest, 1991 (WISC-III - Wechsler intelligence scale for children).

### Variables included

- DO: direct order
- ID: indirect order
- TO: DO+IO
IV Symbol cancelling test, 1985 (Mezulam)
* Variables included:
- number of right answers
- number of errors
- number of omitted answers
- task execution time
V Letter cancelling test, 1985 (Mezulam)

### Variables included

- number of right answers
- number of errors
- number of omitted answers
- task execution time

The assessment of cognitive function adhered to specification defined in the literature (Rey, 1998; Wechsler, 1991; Mezulam, 1985)^25^, ^26^, ^27^.

### Analysis of results

Due to the nature of variables, results were summarized in tables, number of subjects per group, the mean, the standard deviation (SD), minimum, median, maximum and the interquartile interval (IQI).

Comparison between groups was done using the Kruskal-Wallis non-parametric analysis of variance; when results were significant (*), further analysis was done using the Kruskal-Wallis multiple comparisons test.^28^

A 5% probability significance level was adopted for all the tests to reject the null hypothesis.

## RESULTS

The OSAHS group (n=24) and the PS group (n=37) showed statistically significant differences in variables A1 (p=0.001), and the PS group showed statistically significant differences in variables A2, A4, AT and A6 in the Rey Test (p=0.020; p=0.05; p=0.004; p=0.05) compared to the control group (n=20) (Table 1). There were no statistically significant differences between groups in the attention tests (Table 2, Table 3, Table 4, Table 5).

1. Analysis of learning by the Rey test. (Table 1)
2. Analysis of the comparison between groups of attention (Table 2, Table 3, Table 4, Table 5)

**Table 1:** Comparison of the Rey test mean scores in the OSAHS, PS and control groups.

Mean				
Group	OSAHS^a^	PS^b^	control	
Variable	N=24	N=37	N=20	p
A1^c^	39,5	34,9	54,0	0,011*
A2^d^	45,5	33,3	49,7	0,020*
A3^e^	40,4	35,9	51,1	0,065
A4^f^	43,0	32,8	53,8	0,005*
A5^g^	41,9	35,2	50,6	0,058
AT^h^	43,5	32,5	53,7	0,004*
B^i^	36,6	41,2	45,9	0,418
A6^j^	42,8	34,8	50,4	0,050*
A7^k^	40,5	36,4	50,2	0,103
R^l^	42,9	33,3	53,0	0,009*

**Key:** OSAHSa: obstructive sleep apnea hypopnea syndrome; PSb: primary snoring; A1c: number of words recalled after a 1st reading of list A; A2d: number of words recalled after a 2nd reading of list A; A3e: number of words recalled after a 3rd reading of list A; A4f: number of words recalled after a 4th reading of list A; A5g: number of words recalled after a 5th reading of list A; ATh: total number of words recalled: A1 to A5; Bi: number of words recalled after reading list B; A6j: number of list A words recalled spontaneously after reading list B;

A7k: number of list A words recalled spontaneously after 20 minutes; R l: number of correct list A words upon reading the recognition list. Kruskal-Wallis test * p<0.05

**Table 2:** Comparison of the digit subtest mean scores in the OSAHS, PS and control groups.

Mean				
Group	OSAHS^a^	PS^b^	control	
Variable	N=24	N=37	N=20	p
DO^c^	36,3	44,1	40,9	0,427
IO^d^	34,9	39,6	50,8	0,064
TO^e^	34,5	41,7	47,6	0,178

**Key:** OSAHSa: obstructive sleep apnea hypopnea syndrome; PSb: primary snoring, DOc; direct order; IOd: indirect order; TOe: DO+IO Kruskal-Wallis test p<0.05

**Table 3:** Comparison of the coding subtest mean scores in OSAHS, PS and control groups.

Group	n	Mean*	p
OSAHS^a^	24	38,9	0,300
PS^b^	37	38,6	
Control	20	48,1	

**Key:** OSAHSa: obstructive sleep apnea hypopnea syndrome; PSb: primary snoring

Kruskal-Wallis test

**Table 4:** Comparison of the letter cancelling test mean scores in the OSAHS, PS and control groups.

Mean				
Group	OSAHS^a^	PS^b^	control	
Variable	N=24	N=37	N=20	p
Right answers	46,9	36,4	42,5	0,204
Omitted answers	35,0	45,7	41,1	0,188
Errors	39,0	42,2	41,1	0,374
Time	38,8	41,1	43,5	0,805

**Key:** OSAHSa: obstructive sleep apnea hypopnea syndrome; PSb: primary snoring

Kruskal-Wallis test

**Table 5:** Comparison of the symbol cancelling test mean scores in the OSAHS, PS and control groups.

Mean				
Group	OSAHS^a^	PS^b^	control	
Variable	N=24	N=37	N=20	p
Right answers	39,9	38,2	47,5	0,345
Omitted answers	42,1	43,8	34,5	0,345
Errors	39,2	44,1	37,5	0,501
Time	39,9	41,1	42,2	0,953

**Key:** OSAHSa: obstructive sleep apnea hypopnea syndrome; PSb: primary snoring

Kruskal-Wallis test

## DISCUSSION

The negative impact of OSDs on learning, memory and attention in children has been frequently mentioned in medical literature. There have been, however, few studies confirming this association, which makes this theme still a highly controversial topic. This paper compared these parameters in groups of children with OSAHS, PS and children with no OSDs, as diagnosed by polysomnography.

In our study we chose to use classical and already standardized tests. Exclusion criteria sought to eliminate clinical conditions that might interfere on our results.

The learning test (Rey test) involves a sequential and identical repetition of the same stimulus. This test evaluates learning strategies, the ability to retain a new stimulus (immediate memory), the attention tone (attention level), preactivation, susceptibility to interference, and memory recall when the multiple choice test is done.

Our results revealed statistically significant differences in A1 recall between the OSAHS and PS groups and the control group, and statistically significant differences in A2 between the PS group and the control group (Rey test). These results showed that immediate memory and the attention level (that interferes with memory processing) are compromised in groups of patients that have OSDs. Evocation is directly related to the attention level and to recall of information presented once, therefore it is also related to immediate memory. For this reason when we analyzed AT (total number of 5 recalls: A1 to A5), we noted a significant difference between the PS group and the control group, which was shown in the low A1, A2 and A4 scores. The worse performance shown by the PS group in the five lists, compared to the OSAHS and the control groups, suggests that these differences were related to information acquisition, storage or recall after many repetitions of the lists. We believe that this loss may be related not only to intermittent hypoxia, but also to the increased number of awakenings during the night and the resulting sleep fragmentation and decreased REM sleep time, which is necessary for consolidating memories. Blunden et al. suggest that this association may be explained by a combination of the cumulative effect of chronic interruption of the sleep pattern during many years and the simultaneous rapid development of the neuronal synaptic network in children. If findings of a significant association between mild polysomnographic changes in the sleep pattern and decreased cognitive performance are shown to be real, these findings will point to an important function of the sleep pattern in facilitating the development and consolidation of memory and learning in children.5 The analysis of A6 (recall of list A immediately after reading list B) revealed a statistically significant difference between the PS group and the control group. This finding assumed that list B (interference or distracting list) interfered mainly with the ability to maintain the attention level in the PS group, which did not happen in the OSAHS group. When the learning task was related to the ability to spontaneously repeat words that were given previously, we noted that after 20 minutes there was no difference between groups in word recall and memory (A7). The retention level of verbal stimuli after 20 minutes is related to long term memory. Based on the abovementioned findings, we concluded that there was no loss of recorded information with time, suggesting that long term memory was preserved; long term memory storage is obtained by a different mechanism compared to immediate memory. Our findings confirm those of Kaemingk et al., who examined the relation between sleep disordered breathing (SDB) and learning, which they then compared with learning in children with no SDB. Their study involved 149 children aged between 6 and 12 years that underwent intelligence, learning, memory and academic performance assessments. Results revealed a significant decrease in learning and memory in the SDB group, but no difference in intelligence, attention or academic performance.^29^

A few authors have reported a decrease in intellectual abilities,30 but other have found no relation between OSAHS and intelligence in children.^17^, ^29^, ^31^ Findings on memory are also controversial, with some authors reporting decreased memory^16^, ^29^ and others not.17 Lewin et al. noted a mild but significant slowness of mental reasoning in a small sample of children with untreated OSAHS compared with children in which OSAHS was treated and with healthy children in the control group.^31^

Evidence from studies on the treatment of OSAHS offers support to a relation between pediatric OSAHS and cognitive function. Tonsillectomy is effective in treating breathing problems in most children with OSAHS, and appears to help improve academic and intellectual performance and behavior.^15^, ^30^, ^32^ A single study on long term results, published by Gozal and Pope, reported poorer academic performance in currently non-snoring teenagers that were snorers in childhood compared to teenagers that were not snorers when younger.^24^

We believe, as Beebe and Gozal^33^ do, that the mechanism by which OSAHS promotes cognitive morbidity and altered learning in childhood remains unclear. Two potential contributors have received increased attention, namely intermittent hypoxia and sleep fragmentation. Although results in the literature are controversial, when facing our results we feel stimulated to carry forward our line of research, particularly undertaking trials to assess learning, memory and attention in children with OSDs. Our aim is to seek neurological causes and polysomnographic parameters that could explain changes in learning and cognitive function.

## CONCLUSION

Children with OSDs (OSAHS and PS) performed worse in the learning test (Rey test) compared to children in the control group, and children with PS performed worse in the learning test compared to children with OSAHS. Attention tests results were similar between the three groups.
